# Cognitive Dysfunction and Affective Mood Disorder Screening in Patients With Chronic Inflammatory Bowel Disease: Protocol for a Prospective Case-Control Study

**DOI:** 10.2196/50546

**Published:** 2023-10-12

**Authors:** Oliviu Florentiu Sarb, Vitalie Vacaras, Adriana Sarb, Maria Iacobescu, Alina-Ioana Tantau

**Affiliations:** 1 Department of Neuroscience Iuliu Hatieganu University of Medicine and Pharmacy Cluj-Napoca Romania; 2 Department of Internal Medicine Heart Institute Iuliu Hatieganu University of Medicine and Pharmacy Cluj-Napoca Romania; 3 Department of Proteomics and Metabolomics MEDFUTURE Research Center for Advanced Medicine Iuliu Hatieganu University of Medicine and Pharmacy Cluj-Napoca Romania; 4 Department of Internal Medicine 4th Medical Clinic Iuliu Hatieganu University of Medicine and Pharmacy Cluj-Napoca Romania

**Keywords:** gut–brain axis, gut-brain, cognitive dysfunction, affective mood disorders, depression, anxiety, stress, cognitive, cognition, depressive, mood, dementia, protein, proteins, neurotrophic, blood, serum, IBD, inflammatory bowel disease, bowel, gastrointestinal, inflammation, inflammatory

## Abstract

**Background:**

Mild cognitive impairment (MCI) and Alzheimer’s disease (AD) might be more frequent in patients with inflammatory bowel disease (IBD), but the relationship between these 2 entities is yet to be entirely established. Certain blood biomarkers (eg, serum amyloid A [SAA] and serum homocysteine [Hcy], which increase in IBD and MCI; brain-derived neurotrophic factor [BDNF], which decreases in MCI and AD but is not clearly modified in IBD; and S100 calcium-binding protein B [S100B], which increases in the blood-brain barrier and neuronal lesions) might predict the stage of MCI or dementia or progression to a further state. The gut–brain axis (GBA) might be the key to the development of MCI in patients with IBD, along with systemic inflammation and the possible and unknown adverse effects of disease-modifying medication.

**Objective:**

The aim of this study is to investigate whether GBA interactions play a role in MCI development in patients with IBD.

**Methods:**

A case-control study will be conducted on at least 100 patients diagnosed with IBD, matched with 100 healthy individual controls. The matching will include sex, age, and education. Patients will be fully examined, and a full interview and a neurological and cognitive examination will be performed. The primary clinical outcomes will be cognitive test scores (Montreal Cognitive Assessment, Trail Making Test, Digit Symbol Substitution Test, forward and backward digit span testing). Depression, stress, and anxiety screening will also be performed. Blood samples from all participants will be collected, and aliquots will be immediately stored in a biobank. Primary laboratory outcomes will include serum levels of presumed cognitive dysfunction blood biomarkers SAA, Hcy, S100B, and BDNF. Follow-up will be performed at 12, 24, 36, and 48 months.

**Results:**

Data collection started in December 2021 and is ongoing. So far, 53 patients with IBD have been recruited and 50 HC matched. Data collection should end in January 2030. Intermediary analysis will be performed in April 2024. We expect patients with IBD to have lower scores on cognitive testing and a positive correlation between disease length and cognitive impairment level. In addition, the levels of stress, anxiety, and depression should be higher in the IBD group. The serum levels of the 4 biomarkers could correlate or anticorrelate with cognitive scores and serve as predictive factors for MCI or dementia development. A higher level of education, a younger age, the absence of malabsorption, and good disease control might serve as protectors against MCI.

**Conclusions:**

GBA interactions, along with systemic inflammation and the adverse effects of medication, might be a cause of MCI and AD development in patients with IBD. Serum biomarkers could prove cheap and useful predictors of MCI development.

**Trial Registration:**

ClinicalTrials.gov NCT05760729; https://clinicaltrials.gov/study/NCT05760729

**International Registered Report Identifier (IRRID):**

DERR1-10.2196/50546

## Introduction

### Background

Inflammatory bowel disease (IBD) is a group of chronic conditions characterized by intestinal inflammation. The 2 most common types of IBD are Crohn’s disease (CD) and ulcerative colitis (UC) [1,2]. The exact mechanisms underlying the development of IBD are yet to be fully understood, but they are thought to be the result of a combination of genetic, environmental, and immunological factors. IBD is believed to be caused by an abnormal immune response that first appears in the gut, where the body’s immune system mistakenly attacks the antigens of the healthy gut bacteria, leading to intestinal inflammation and damage to the intestinal barrier. Exposure to certain environmental triggers, such as a diet high in animal fats, smoking, and, most of all, bacterial or viral infections, may increase the risk of IBD. It is believed that the interplay between these factors leads to a self-perpetuating cycle of inflammation and tissue damage, which contributes to the chronic nature of IBD (a so-called vicious cycle). Systemic inflammation plays a role in IBD pathogenesis, which is proven by the beneficial effect of certain anti-inflammatory medications [3].

Clinical manifestations include common digestive symptoms, such as abdominal pain and cramping, diarrhea (which might be bloody), rectal bleeding, weight loss, fatigue, loss of appetite, anemia, nausea and vomiting, dehydration, and fever. In addition to these digestive symptoms, patients with IBD may also experience extraintestinal manifestations, such as joint pain, skin rashes, eye inflammation, and liver disease. The severity and frequency of symptoms can vary greatly between individuals and can also change over time in the same individual. CD carries a higher risk of extraintestinal manifestations, often believed to be caused by one of its pathogenic mechanisms or malabsorption [1,2].

Mild cognitive impairment (MCI) refers to a decline in cognitive abilities that is greater than that in normal aging but certainly not severe enough to interfere with daily life. MCI is often considered a transitional stage between normal aging and more severe forms of cognitive decline, such as dementia. MCI is most commonly characterized by memory problems, such as difficulty in recalling names and recent events, but it can also involve problems with other cognitive abilities, such as language, decision-making, and attention. The impairment of daily activities is a key criterion to diagnosing dementia and the difference between MCI and dementia [4]. Some of the known risk factors for MCI include age, family history, certain medical conditions, lifestyle factors (eg, smoking and excessive alcohol consumption), and certain medications [4]. There are some modern theories that imply inflammation as a mechanism underlying cognitive impairment [5-8]. A diagnosis is typically made based on a thorough medical and neurological evaluation, as well as a comprehensive assessment of the individual’s cognitive abilities. This may involve a battery of tests, such as memory and language tests, as well as brain imaging studies. The most important thing to do when a cognitive impairment is suspected is follow-up over time, as early intervention might stop the progression of the disease [4]. MCI can sometimes progress to Alzheimer’s disease (AD) or other types of dementia, depending on the cause, age, and other factors [9]. AD is a progressive brain disorder that affects memory, thinking, and behavior. It is the most common cause of dementia. The symptoms of AD usually start with mild memory loss but eventually progress to affect thinking, communication, and the ability to perform daily activities. As the disease progresses, patients may become increasingly dependent on others for their care [10]. Some studies have revealed a link between IBD and the development of AD [6,7,11,12]. Vascular dementia is a type of dementia that occurs due to problems in the blood supply to the brain. It is caused by damage to the blood vessels that deliver oxygen and nutrients to the brain, leading to brain injury and reduced brain function. It is often caused by conditions such as stroke, high blood pressure, and heart disease. Risk factors for vascular dementia include age, smoking, diabetes, and a family history of stroke or heart disease [13].

“Stress disorder” is a term used to describe a mental health condition that can develop in response to a traumatic or stressful event. Individuals may experience a range of symptoms, including intrusive thoughts or memories of the traumatic event; nightmares; and avoidance of people, places, or activities that are associated with the trauma. They may also experience physical symptoms, such as a racing heart, sweating, and muscle tension [14]. “Anxiety disorder” is a general term that refers to a group of mental health conditions characterized by excessive and persistent feelings of worry, fear, and unease [15]. Depression is a mental health condition characterized by persistent feelings of sadness, hopelessness, and a loss of interest in activities that were once enjoyable. It is a common and serious condition that can have a significant impact on an individual’s daily life and overall well-being. In addition to feelings of sadness, depression can also cause a range of physical and emotional symptoms, including fatigue, changes in appetite and sleep patterns, difficulty in concentrating, and thoughts of self-harm or suicide. The exact cause of depression is not fully understood, but it is believed to be related to a combination of genetic, biological, environmental, and psychological factors [16-18].

Homocysteine (Hcy) is an amino acid. Studies have shown that individuals with IBD have higher serum concentrations of Hcy compared to healthy controls (HC). The exact reason for this association is not fully understood, but it may be related to nutrient deficiencies caused by the inflammation and malabsorption in the gut that often occur in IBD. There is evidence to suggest that elevated levels of Hcy in the blood are associated with an increased risk of MCI and dementia. High Hcy levels have been linked to damage of the blood vessels in the brain, leading to decreased blood flow and oxidative stress, both of which can contribute to cognitive decline [19-23].

Serum amyloid A (SAA) is an acute-phase protein that is produced by the liver in response to inflammation. Amyloid proteins are abnormal fibrous proteins that can build up in tissues and organs, leading to the formation of amyloid plaques. In some cases, amyloid proteins can also enter the bloodstream and circulate in the body. Elevated levels of SAA can be an indicator of various diseases, such as AD, systemic amyloidosis, and hereditary transthyretin amyloidosis. Studies have shown that SAA can induce neuroinflammation and damage neurons in the brain, which may contribute to the development and progression of AD. In addition, SAA has been found to accumulate in the brain in the form of amyloid plaques, which are a hallmark feature of AD. There is limited research on the relationship between SAA levels and IBD. Some studies have suggested that individuals with IBD may have elevated levels of amyloid proteins in their bloodstream, possibly as a result of chronic inflammation in the gut [24-29].

Brain-derived neurotrophic factor (BDNF) is a type of protein that is important for the growth, maintenance, and survival of neurons in the central nervous system. BDNF plays a key role in processes such as neuroplasticity, learning, and memory formation. Low levels of BDNF have been associated with several neurological and psychiatric disorders, including depression and AD. BDNF plays a role in the inflammation associated with IBD. BDNF levels are modified in IBD, but there is little evidence of the direction [30-33].

S100 calcium-binding protein B (S100B) is a protein that is found in a variety of tissues, including the brain, where it has been implicated in several neurobiological processes, including synaptic plasticity, neurogenesis, and neuroinflammation. High serum S100B levels have been associated with a variety of neurological disorders, including traumatic brain injury, stroke, and neurodegenerative diseases. Studies have shown that S100B levels are elevated in the blood and intestinal tissue of patients with IBD, suggesting that the protein may play a role in the development and progression of the disease. In addition, high levels of S100B have been associated with increased inflammation and oxidative stress [34].

In conclusion, IBD is a combination of intestinal barrier dysfunction and intestinal and systemic inflammation. Due to intestinal barrier dysfunction, the intestinal microbiome is affected in the course of the disease or it is the cause of the disease (IBD). Several studies have shown the relationship between the gut–brain axis (GBA) and the development of depression, affective mood disorders, and stress, which might promote the development of cognitive impairment. Systemic inflammation is known to increase cardiovascular risk, which is a risk factor for cognitive impairment. Other risk factors, such as smoking, alcohol consumption, and other diseases, also promote the development of cognitive impairment. Some studies have suggested a link between IBD and cognitive disorders, while other studies have indicated a link between mood disorders and IBD, and still others have suggested a link between mood disorders and cognitive disorders. Therefore, this might be a vicious cycle.

**Figure 1:**
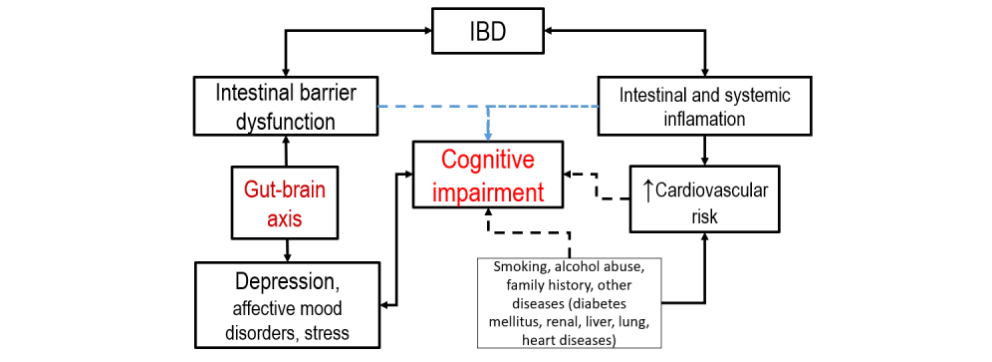
Possible mechanisms underlying the development of cognitive impairment associated with IBD. IBD: inflammatory bowel disease.

In this study, blood biomarkers, such as SAA (an inflammatory biomarker, a precursor of beta-amyloid plaques in the brain), Hcy (a pro-inflammatory molecule increased in cognitive disorders), S100B (a neuronal structural protein increased in brain injury), and BDNF (decreased in cognitive disorders) were selected in order to validate the hypothesis that these biomarkers could be used as predictive factors for cognitive impairment associated with IBD. As a summary of the basic knowledge that led to the idea of this study, Figure 1 shows the possible implied mechanisms underlying the development of MCI in patients with chronic IBD.

### Aim

The aim of this study is to investigate whether GBA interactions play a role in the development of MCI in patients diagnosed with IBD, mainly due to pathological changes at the intestinal level (destruction of the intestinal barrier and pathogenic species in the intestinal microbiome). Presumed cognitive dysfunction blood biomarkers (SAA, Hcy, S100B, BDNF) will be used as predictive factors for the development of MCI or dementia in the study groups. The primary and secondary objectives of the study can be reviewed in Table 1.

**Table 1:** Primary and secondary objectives.

Objective type	Description
Primary	To describe the occurrence of MCI^a^ in patients with MCI compared to HC^b^ To determine cognitive tests scores for the IBD^c^ group compared to HC To determine serum levels of SAA^d^, Hcy^e^, BDNF^f^, and S100B^g^ protein and their association with cognitive testing scores compared to HC To determine whether there is any correlation between IBD duration, type, blood biomarkers and cognitive scores
Secondary	To describe the progression of MCI during the follow-up period in both groups To assess the occurrence of stress and anxiety disorders in the IBD group compared to HC To assess the occurrence of depression in the IBD group compared to HC To identify novel biological markers that can predict the stage of MCI or its progression in both groups To quantify the quality of life of both groups To identify risk factors for the development of MCI in both groups To identify the comorbidities of participants in both groups To quantify the clinical activity scores of the IBD group during patient interviews

^a^MCI: mild cognitive impairment.

^b^HC: healthy controls.

^c^IBD: inflammatory bowel disease.

^d^SAA: serum amyloid A.

^e^Hcy: homocysteine.

^f^BDNF: brain-derived neurotrophic factor.

^g^S100B: S100 calcium-binding protein B.

## Methods

### Study Design

The study will be a single-center, prospective, observational, analytic study with a group of at least 100 patients with a diagnosis of certainty of IBD and a group of at least 100 HC. The study is part of a PhD thesis, and funding was obtained from the parent university.

### Sites, Population, and Case and Control Definitions

The study will take place in Cluj-Napoca, Romania, in the Clinical CF Hospital and the Regional Institute for Gastroenterology and Hepatology “Octavian Fodor.” An agreement was obtained from all the participant clinics.

Two separate groups consisting of at least 100 patients with IBD and at least 100 HC will be recruited. The study will be carried out from May 2021 to January 2030. The HC will be matched with the patients with IBD based on age, sex, and education.

Patients included in the case (IBD) group need to have a clear diagnosis of IBD. For this, a histopathological examination proof will be obtained from the databases of the participant centers or from the personal records of the patients (if the site does not have the original proof diagnosis).

The HC will be recruited from the day care hospital and ambulatory. Their diagnosis must have never been IBD, and they should be in good health at the moment of inclusion.

The inclusion criteria are listed in Table 2 and the exclusion criteria in Table 3.

**Table 2:** Inclusion criteria.

Study group	Inclusion criteria
IBD^a^	Males and females aged over 18 years Patients with a confirmed IBD diagnosis Written informed consent obtained
HC^b^	Males and females aged over 18 years Good level of health Written informed consent obtained

^a^IBD: inflammatory bowel disease.

^b^HC: healthy controls.

**Table 3:** Exclusion criteria.

Exclusion criteria		Details
**Absolute exclusion criteria**		
	Prior stroke or myocardial infarction or cardiac arrest	Patients with known stroke and myocardial infarction are already known to have an accelerated cognitive decline rate due to a vascular mechanism. Postanoxic encephalitis after cardiac arrest carries an additional risk of cognitive decline.
	Severe organ failure	This includes the presence of end-stage respiratory, liver, renal, and cardiac diseases.
	Familial AD^a^	Patients with known familial AD develop the disease at an earlier age.
	Concomitant past and current neurological disorders	These include epilepsy, encephalopathy due to any cause, history of severe head trauma, history of encephalitis, multiple sclerosis, Parkinson’s disease, brain tumor, and dementia due to any cause.
	Pregnancy	Pregnant women will not be included because they carry potentially other risks and might be stressed about their situation.
	Prior psychiatric disorders and chronic use of neuroleptics and anticholinergic medication	These include all psychiatric pathologies, including depression, anxiety disorder, attention deficit hyperactivity disorder, autism spectrum disorders, posttraumatic stress disorder, schizophrenia, personality disorders, and alcohol and drug abuse. Also in this category is the use of neuroleptics, which are drugs that decrease cerebral function, although they might interfere with cognitive testing, depending on dose. Anticholinergic medication might slow cognitive functioning.
	Unclear diagnosis	Patients with an unclear diagnosis will not be recruited as they might have a different pathology.
	Involvement in clinical trials	Participation in clinical trials for medication might interfere with the study results because of the unknown side effects of the medication or other factors.
	Current and past history of neoplasia	Patients with neoplasms already have a poor performance status, and this might negatively influence the results of this study. in addition, the medication might interfere with cognitive testing.
	Use of vitamin B12 and B9 supplements	Vitamin B12 and B9 supplements might decrease serum levels of Hcy^b^.
	Short bowel syndrome	Intestinal resection carries the risk of malabsorption and might be a cause of cognitive impairment.
	Diabetes mellitus	Diabetes mellitus carries an additional risk of MCI^c^.
	Hypothyroidism	Hypothyroidism leads to decreased brain performance.
	Prior diagnosis of atrial fibrillation	Patients with known atrial fibrillation are at greater risk of cognitive dysfunction due to microembolic events.
	Uncontrolled arterial hypertension	Patients with uncontrolled hypertension carry an additional risk of cognitive dysfunction.
**Relative exclusion criteria**		
	Acute IBD^d^ flares	Acute flares involve a burden of stress and anxiety, which might lead to false-positive results in cognitive testing. Patients will be informed of the possibility of inclusion, and if they agree, testing will be performed after the acute flare is over.
	Age over 70 years	Patients aged over 70 years might have age-related cognitive impairment, which might interfere with the study results.

^a^AD: Alzheimer’s disease.

^b^Hcy: homocysteine.

^c^MCI: mild cognitive impairment.

^d^IBD: inflammatory bowel disease.

### Study Timeline

Patients will be recruited based on the inclusion and exclusion criteria. The recruitment moment will be called “baseline.” Follow-up will be performed at 12, 24, and 36 months. A systematization of the procedures is presented in Table 4.

**Table 4:** Study timeline.

Objective	Timeline			
	Baseline	12-month follow-up	24-month follow-up	36-month follow-up
Eligibility assessment	X^a^	—^b^	—	—
Signing of informed consent form	X	—	—	—
Exclusion and inclusion criteria reassurance	X	X	X	X
Demographics	X	—	—	—
Relationship status	X	—	—	—
Short cognitive and affective questionnaire	X	X	X	X
Physical examination	X	X	X	X
Medication assessment	X	X	X	X
Medical history assessment	X	—	—	—
Collection of blood sample and storage	X	—	—	—
Detailed risk factor questionnaire	X	—	—	—
Clinical disease activity score	X	X	X	X
Depression, stress, and anxiety screening	X	X		
COVID-19 questionnaire	X	—	—	—
Quality-of-life questionnaire	X	X	X	X
MOCA^c^	X	X	X	X
MIS^d^	X	X	X	X
TMT^e^-A	X	—	—	X
TMT-B	X	—	—	X
DSST^f^	X	—	—	X
FDST^g^	X	—	—	X
BDST^h^	X	—	—	X
ADL^i^ questionnaires	X	X	X	X
IADL^j^	X	X	X	X

^a^X: applicable.

^b^—: not applicable.

^c^MOCA: Montreal Cognitive Assessment.

^d^MIS: memory impairment score.

^e^TMT: Trail Making Test.

^f^DSST: Digit Symbol Substitution Test.

^g^FDST: forward digit span testing.

^h^BDST: backward digit span testing.

^i^ADL: activities of daily living.

^j^IADL: Instrumental Activities of Daily Living.

### Procedures and Examinations

Clinical procedures and examinations will be performed in a silent room so there is no disturbance to both examiner and participant. Participants will receive information about each test that will be conducted. The scales and questionnaires used in the study are listed in Table 5.

**Table 5:** Scales used in the study.

Objective	Scales
Assessment of IBD^a^ severity	HBI^b^ for patients with CD^c^ and SCCAI^d^ for patients with UC^e^
Assessment of quality of life	Romanian WHOQOL-BREF^f^ questionnaire
Assessment of depression, anxiety, and stress	Romanian DASS^g^-21
Cognitive assessment	Romanian MOCA^h^ 7.1, TMT^i^-A and TMT-B, backward digit testing, forward digit testing, DSST^j^
Assessment of COVID-19	Short COVID-19 questionnaire
Assessment of ADL^k^	IADL^l^ scale, Katz ADL scale

^a^IBD: inflammatory bowel disease.

^b^HBI: Harvey-Bradshaw Index.

^c^CD: Crohn’s disease.

^d^SCCAI: Simple Clinical Colitis Activity Index.

^e^UC: ulcerative colitis.

^f^WHOQOL-BREF: World Health Organization Quality of Life Brief Version.

^g^DASS: Depression-Anxiety-Stress Scale.

^h^MOCA: Montreal Cognitive Assessment.

^i^TMT: Trail Making Test.

^j^DSST: Digit Symbol Substitution Test.

^k^ADL: activities of daily living.

^l^IADL: Instrumental Activities of Daily Living.

The Montreal Cognitive Assessment (MOCA) 7.1 scale includes the Trail Making Test (TMT), drawing, long-term memory testing, verbal fluency tests, short-term memory recall testing, calculus, and spatial and temporal orientation questions. The maximum score obtained is 30 points. The normal range is ≥26 points. An additional point is given to patients who have an education level of ≤12 years.

The memory impairment score (MIS) is derived from the MOCA scale. The maximum score is 15 points. It derives from the short-term memory recall test. Subjects have to recall 5 words to gain points. If they recall the words spontaneously, 3 points are awarded for each word. If any clue is provided, 2 points are awarded if the word is recalled. If variants are provided, 1 point is awarded if the word is recalled. No point is awarded if the word is not recalled.

The TMT involves the subject drawing a trail between 2 points, with the entire operation being timed. It has 2 different parts, TMT-A and TMT-B. TMT-A involves drawing a continuous line between numbers. TMT-B involves drawing a continuous line, alternating letters and numbers. An example of the test is provided before each part. TMT-A has a maximum time of 100 seconds, and 101 seconds means the subject has failed the test. TMT-B has a maximum time of 300 seconds, and 301 seconds means the subject has failed the test.

The Digit Symbol Substitution Test (DSST) involves the subject changing some numbers to symbols as specified in the legend of the test. A training zone is first created. If the training is completed, the subject is given 90 seconds to complete as many substitutions as possible. Correct and incorrect answers are counted.

In forward digit span testing (FDST) and backward digit span testing (BDST), the subject must recall the numbers provided by the examiner in forward order and then in backward order. FDST involves strings of numbers ranging from 2 to 8 numbers, and BDST involves strings of numbers ranging from 2 numbers to 7 numbers. Each string has 2 different variants, and the subject must make a maximum of 1 mistake in these variants, otherwise the test ends.

The Depression-Anxiety-Stress Scale (DASS) is a scale that includes 21 questions split into 3 categories (depression, stress, and anxiety), with 7 questions per category, the questions being interleaved. Each question has a score from 0 to 3, and the result is multiplied by 2.

The Harvey-Bradshaw Index (HBI) and the Simple Clinical Colitis Activity Index (SCCAI) are used for screening the subject. In this study, they are part of the inclusion criteria.

The quality-of-life instruments available in Romania are limited; therefore, the World Health Organization Quality of Life Brief Version (WHOQOL-BREF) will be used in order to determine the quality of life of our participants. The Instrumental Activities of Daily Living (IADL) is an 8-item scale that includes some instrumental daily activities, and it is a useful and essential tool for differentiating MCI from dementia. The Katzl ADL scale is a 6-item scale that includes casual daily activities and is also a useful and essential tool for differentiating MCI from dementia.

A blood sample will be obtained from each participant.

### Outcomes

The first to be mentioned are demographic variables, which will be useful for stratifying our participants; the expected answers are presented in Table 6.

**Table 6:** Demographic variables.

Demographic characteristics	Expected answers
Education level	Absolute number of years
Current living environment	Rural, urban <10,000 inhabitants, urban 10,000-49,999 inhabitants, urban 50,000-99,999 inhabitants, urban >100,000 inhabitants
Marital status	Married, single, cohabitation, divorced, polygamy, widowed
Employment	Employed full-time, employed part-time, freelancer, retired, student, trainee
Toxic working environment	Yes, no, do not know
Night shifts	Yes, no
Financial status	Enough for a living and saving, enough for a living but not saving, not enough for a living
Monthly wage (average per family member)	<1500 lei (<USD 316.31), 1500-2500 lei (USD 316.31-527.18), 2500-3500 lei (USD 527.18-738.05), 3500-4500 lei (USD 738.05-948.93), >4500 lei (>USD 948.93)

The relationship status is important for affective mood but also for cognitive functioning; therefore, 3 questions will be answered by the participants, listed in Table 7.

**Table 7:** Current and past relationship data.

Question	Expected responses
Married before diagnosis of IBD^a^	Yes, no, not available
Relationship destabilization due to the disease	Yes, and it caused a broken relationship or divorce; yes, but it did not cause a broken relationship or divorce; no
Helped with the disease by the life partner	Yes, no, not available

^a^IBD: inflammatory bowel disease.

A short cognitive assessment will be performed in order to accommodate the participants in the subsequent questionnaires and tests. Questions are mentioned in Table 8.

**Table 8:** Short cognitive (long-term memory) and affective assessment.

Question	Expected responses
Trouble with memory in the past month	Yes, occasionally, no
Changes in personality	Yes, no
Difficulties in handling daily activities due to memory changes	Yes, no
Family history of dementia	Yes, AD^a^; yes, other dementia; no; do not know
Current president of Romania	Correct or incorrect answer
The year of the Great Union in Romania	Correct or incorrect answer
Naming a pencil	Correct or incorrect answer
Naming a medical coat	Correct or incorrect answer
Naming a clock	Correct or incorrect answer

^a^AD: Alzheimer’s disease.

A short clinical examination will be performed. A full history of IBD will be taken. Variables obtained are mentioned in Table 9. The HBI and SCCAI will be the clinical disease activity scales used in the study, as mentioned in Table 10. Risk factor assessment will also be performed (Table 11).

**Table 9:** Physical examination and past medical history.

Variables		Expected responses
**Physical examination**		
	Blood pressure	Value (mmHg)
	Pulse	Absolute number (beats/minute)
	Respiratory rate	Absolute number (respirations/minute)
	Peripheric oxygenation percentage	Value (%)
Medication assessment		All concomitant medications used (international common names and dosing and posology)
**IBD^a^ history assessment**		
	Year of diagnosis	Absolute number
	Montreal classification	A-L-B^b^ for CD^c^ patients, E-S^d^ for UC^e^ patients
	Family history of IBD	Yes, no, do not know
	Number of flares in the past 12 months	Absolute number
	Number of flares since diagnosis	Absolute number
	Number of hospitalizations for flares in the past 12 months	Absolute number
	History of corticosteroid dependency	Yes, no
	Duration of corticosteroid dependency (if the above answer is yes)	Absolute number (months)
	Current medication	Name, dose, duration
	Past medication	Name, dose, duration
	Complications	Enumeration

^a^IBD: inflammatory bowel disease.

^b^A-L-B: onset (A), disease location (L), and disease behavior (B).

^c^CD: Crohn’s disease.

^d^E-S: extent (E) and severity (S).

^e^UC: ulcerative colitis.

**Table 10:** Clinical disease activity score assessment.

Scales and items			Scoring (points in parentheses)
**HBI^a^ (0-4 points=remission; 5-7 points=mild disease; 8-16 points=moderate disease; >16 points=severe disease)**			
	General health	Very well (0), slightly below par (1), poor (2), very poor (3), terrible (4)	
	Abdominal pain	None (0), mild (1), moderate (2), severe (2)	
	Liquid or soft stools on the previous day	Absolute number (1 point for each)	
	Abdominal mass	None (0), dubious (1), definite (2), definite tender (3)	
	Complications	Arthralgia (1), uveitis (1), erythema nodosum (1), aphthous ulcer (1), pyoderma gangrenosum (1), anal fissure (1), new fistula (1), abscess (1)	
**SCCAI^b^ (0-20 points; ≥5=active disease)**			
	Bowel frequency	0-3 stools (0), 4-6 stools (1), 7-9 (stools 2), >9 stools (3)	
	Bowel frequency (night)	0 stools (0), 1-3 stools (1), 4-6 stools (2)	
	Urgency defecation	No urgency (0), hurry (1), immediately (2), incontinence (3)	
	Blood in stool	No blood (0p, traces (1), occasionally frank (2), usually frank (3)	
	General health	Very well (0), slightly below par (1), poor (2), very poor (3), terrible (4)	
	Extracolonic manifestations	No feature (0), 1 feature (1), 2 features (2), 3 features (3), 4 features (4), 5 features (5)	

^a^HBI: Harvey-Bradshaw Index.

^b^SCCAI: Simple Clinical Colitis Activity Index.

**Table 11:** Risk factor questionnaire.

Detailed risk factor questionnaire	Expected responses
Active smoker status	Yes, no, ex-smoker
Number of smoking years	Absolute number
Number of abstinence years	Absolute number
Daily number of cigarettes	Absolute number
Alcohol drinking rate	Daily, 3-5 times per week, 1-2 times per week, rarer, no drinking, other options (to be mentioned)
30 minutes of physical activity	Daily, 5 times per week, rarer, no physical activity, other frequency (to be mentioned)
Dietary trend	Nonspecific diet, vegetarian, vegan, flexitarian, colitis regimen, other (to be mentioned)
Number of sleeping hours per day (24 hours)	Absolute number
Daytime tiredness	Yes, no
Troubles with maintaining sleep	Yes, no
Troubles with falling asleep	Yes, no

A cognitive assessment must always be preceded by a screening of depression, anxiety, and stress because this may falsely create a diagnosis of cognitive dysfunction. Scales and their interpretations can be seen in Table 12.

**Table 12:** Depression, anxiety, and stress screening; COVID-19 impact; current quality of life; and ADL^a^ assessment.

Scales and items			Expected responses
DASS^b^-21 scale (7 items each for depression, anxiety, and stress)			Depression (0-9, 10-13, 14-20, 21-27, ≥28 points), anxiety (0-7, 8-9, 10-14, 15-19, ≥20 points), stress (0-14, 15-18, 19-25, 26-33, ≥34 points)
**COVID-19 impact questionnaire**			
	Vaccination status	Yes, no	
	COVID-19 vaccine (if the above answer is yes)	BNT162b2, NJ-78436735 (Ad26.COV2.S), AZD1222 (ChAdOx1), mRNA^c^-1273, other (mentioned in the questionnaire)	
	Number of doses	Absolute number	
	History of infection	Yes, no, do not know	
	WHOQOL-BREF^d^	Social relationships (4-20 points), environment (4-20 points), physical health (4-20 points), psychological (4-20 points)	
**ADL**			
	Katz ADL	0-6 points	
	IADL^e^	0-8 points	

^a^ADL: activities of daily living.

^b^DASS: Depression Anxiety Stress Scale.

^c^mRNA: messenger ribonucleic acid.

^d^WHOQOL-BREF: World Health Organization Quality of Life Brief Version.

^e^IADL: Instrumental Activities of Daily Living

Cognitive assessment will include the baseline scales and follow-up at 12 months. The scoring is shown in Table 13.

**Table 13:** Cognitive assessment scoring.

Timeline and scales			Scoring
**Baseline**			
	MOCA^a^	0-30 points: executive function (0-5 points), naming (0-3 points), memory (0-5 points), attention (0-6 points), calculus (0-3 points), language (0-3 points), orientation (0-6 points), abstractization (0-2 points)	
	MIS^b^	0-15 points	
	BDST^c^	0-14 points	
	FDST^d^	0-16 points	
	TMT^e^-A	0-100 seconds (101 seconds means failed test)	
	TMT-B	0-300 seconds (301 seconds means failed test)	
	DSST^f^	Absolute number of correct answers (maximum 90 correct answers), absolute number of incorrect answers (maximum 90 incorrect answers)	
**12-month follow-up**			
	MOCA	0-30 points: executive function (0-5 points), naming (0-3 points), memory (0-5 points), attention (0-6 points), calculus (0-3 points), language (0-3 points), orientation (0-6 points), abstractization (0-2 points)	
	MIS	0-15 points	
**24-month follow-up**			
	MOCA	0-30 points: executive function (0-5 points), naming (0-3 points), memory (0-5 points), attention (0-6 points), calculus (0-3 points), language (0-3 points), orientation (0-6 points), abstractization (0-2 points)	
	MIS	0-15 points	
**36-month follow-up**			
	MOCA	0-30 points: executive function (0-5 points), naming (0-3 points), memory (0-5 points), attention (0-6 points), calculus (0-3 points), language (0-3 points), orientation (0-6 points), abstractization (0-2 points)	
	MIS	0-15 points	
**48-month follow-up**			
	MOCA	0-30 points: executive function (0-5 points), naming (0-3 points), memory (0-5 points), attention (0-6 points), calculus (0-3 points), language (0-3 points), orientation (0-6 points), abstractization (0-2 points)	
	MIS	0-15 points	
	BDST	0-14 points	
	FDST	0-16 points	
	TMT-A	0-100 seconds (101 seconds means failed test)	
	TMT-B	0-300 seconds (301 seconds means failed test)	
	DSST	Absolute number of correct answers (maximum 90 correct answers), absolute number of incorrect answers (maximum 90 incorrect answers)	

^a^MOCA: Montreal Cognitive Assessment.

^b^MIS: memory impairment score.

^c^BDST: backward digit span testing.

^d^FDST: forward digit span testing.

^e^TMT: Trail Making Test.

^f^DSST: Digit Symbol Substitution Test.

### Laboratory Procedures and Outcomes

We will obtain the serum from 5 mL of the blood sample of each participant and quantitatively measure serum SAA, Hcy, S100B, BDNF levels. The blood samples will be obtained at baseline. Other blood measurements will be performed at the hospital’s laboratory as part of the visit, and values will be taken from the registry. All the necessary data that will be obtained from blood testing are mentioned in Table 14.

**Table 14:** Blood sample outcomes.

Markers		Measurement unit
**Presumed cognitive biomarkers (serum levels)**		
	SAA^a^	mcg/mL
	Hcy^b^	µmol/L
	BDNF^c^	ng/mL
	S100B^d^	mcg/mL
**Common biomarkers (taken from the hospital registry from the baseline presentation period)**		
	C-reactive protein	mg/dL
	Total hemoglobin	g/dL
	Red blood cell count	number/mm^3^
	Fecal calprotectin	μg/mg
	Triglycerides	mg/dL
	Total cholesterol	mg/dL
	Low-density lipoprotein (LDL) cholesterol	mg/dL
	High-density lipoprotein (HDL) cholesterol	mg/dL
	LDL/HDL ratio	fraction
	Neutrophile-to-lymphocyte (NLR) ratio	fraction
	Erythrocyte sedimentation rate	mm/hour
	Mean corpuscular volume (MCV)	%
	Basal blood glucose	mg/dL
	Fibrinogen	mg/dL
	Lactate dehydrogenase (LDH)	IU/L
	Creatine kinase (CK)	U/L
	CK-MB	% out of total CK

^a^SAA: serum amyloid A.

^b^Hcy: homocysteine.

^c^BDNF: brain-derived neurotrophic factor.

^d^S100B: S100 calcium-binding protein B.

#### Preparation of Serum Samples

The required materials are a serum collection vaccutainer, a fixed-angle centrifuge for 15 mL tubes, 1.5 mL protein low-binding tubes, 100-1000 µL tips, and a cryogenic box.

For serum preparation, after the blood is collected, the tube will be inverted gently, as indicated in the manufacturer’s instructions (in general 5-6 times). The tube will be then left, as indicated in the manufacturer’s instructions (in general 30 minutes), at room temperature to form a blood clot. Next, the tube will be centrifuged at 2000× *g* for 10 minutes at 4 °C, as indicated in the manufacturer’s instructions, and 500 µL of serum will be aliquoted in 1.5 mL protein low binding tubes. The serum aliquots will be immediately frozen at –80 °C until use. No more than 2 freeze-thaw cycles will be allowed. The tube with the blood clot will be discarded in a biohazard waste container.

#### Absolute Quantification of Biomarkers

SAA, Hcy, S100B, and BDNF are biomarkers that will be quantified from the serum samples using standardized solid-phase sandwich enzyme-linked immunosorbent assay (ELISA) kits. Table 15 presents the kits that will be used and their related characteristics.

**Table 15:** ELISA^a^ kits to be used for biomarker quantification.

Biomarker	ELISA kit	Serum volume required (µL)	Kit performance	Assay duration (hours)
SAA^b^	Abclonal catalog # RK04228 or similar	100	Range:0.156-10 ng/mL Sensitivity: <0.071 ng/mL Specificity: 50 ng/mL Intra-assay precision: CV^c^<10% Interassay precision: CV<12%	5.0
Hcy^d^	Assaygenie catalog # HUFI04768 or similar	50	Range: 7.813-500 pmol/mL Sensitivity: <4.688 pmol/mL Specificity: 50 ng/mL Intra-assay precision: CV<8% Interassay precision: CV<10%	2.5
S100B^e^	Abclonal catalog # RK02234 or similar	100	Range: 46.9-3000 pg/ml Sensitivity: <23.5 pg/mL Specificity: 50 ng/mL Intra-assay precision: CV<10% Inter-assay precision: CV<15%	5.0
BDNF^f^	Abclonal catalog # RK00074 or similar	100	Range: 23.4-1500 pg/ml Sensitivity: <6.3 pg/mL Specificity: 50 ng/mL Intra-assay precision: CV<10% Interassay precision: CV<15%	5.0

^a^ELISA: enzyme-linked immunosorbent assay.

^b^SAA: serum amyloid A.

^c^CV: coefficient of variation.

^d^Hcy: homocysteine.

^e^S100B: S100 calcium-binding protein B.

^f^BDNF: brain-derived neurotrophic factor.

The required materials for this procedure are the serum aliquots, ice, a fixed-angle centrifuge for 2 mL tubes, 1.5 mL protein low-binding tubes, tips, pipettes, 15 or 50 mL tubes (depending on the ELISA kit instructions), a 96-well plate shaker (if mentioned by the ELISA kit manufacturer), a 96-well plate mixer with heating (if mentioned by the ELISA kit manufacturer), and a multiplate reader.

Serum aliquots will be thawed on ice and then mixed gently. Samples will be centrifuged at 13000× *g* for 2 minutes at 4°C to remove the cell debris. The volume indicated in the kit manufacturer’s protocol will be taken. The instructions will be used for sample preparation and analysis. Measurements will be made in duplicate.

#### Calculation of Results

The optical density (OD) of each well will be read using a microplate reader set to 450 nm. The duplicate readings for each standard, control, and sample will be averaged, and the average 0 standard OD will be subtracted. A standard curve will be created by reducing the data using computer software capable of generating a 4-parameter logistic (4-PL) curve fit. Sample concentrations will be calculated from the equation. If samples have been diluted, the concentration read from the standard curve will be multiplied by the dilution factor.

### Power Calculation, Statistical Model, and Data Analysis

Based on previous studies, the power of the study, with an α of .05 and a power of 80%, the required number of cases and controls is 52 and 26, respectively, for a mean difference in 2 points (SD 3.0) in MOCA scales.

Statistics will be computed using IBM SPSS software version 28.0.1 or newer. Descriptive analysis of the baseline characteristics of the overall population by gender, age, and education will be conducted. Continuous variables will be presented as the mean (SD) or the median (IQR), and countable data will be presented as numbers and percentages. Comparisons of groups will be conducted using the t-test or the Wilcoxon rank-sum test based on the type of distribution of data and the homogeneity of the variance. The chi-square test or the Fisher exact probability method will be used for counting data, as appropriate.

Multiple linear and logistical regression analyses will be performed to evaluate the association between outcomes and potential risk factors identified in the questionnaires. Participants lost to follow-up will be treated as right-censored data. The Pearson coefficient will be used to assess the correlation between cognitive scores and serum levels of the studied biomarkers. A 2-sided significance level of .05 will be used for all primary and secondary analyses unless stated otherwise.

### Ethical Considerations

The protocol was approved by the Ethics Committees of the Iuliu Hatieganu University of Medicine and Pharmacy, Cluj-Napoca (# 52/28.02.2022), the Regional Institute of Gastroenterology and Hepatology “Octavian Fodor” (# 10148/ 11.10.2022), and the Clinical CF Hospital (# 14/13.07.2022). It was registered with ClinicalTrials (identifier NCT05760729). The study will be conducted in accordance with the ethical principles outlined in the current version of the Declaration of Helsinki of 1975, revised in 2013, and all applicable local regulatory requirements.

All participants will be required to personally sign and date the latest approved version of the informed consent form (ICF) before any study specific procedures are performed. A written version of the participant information and informed consent will be presented to each participant, detailing no less than the exact nature of the study, what it will involve for the participant, the implications and constraints of the protocol, the known side effects and any risks involved in taking part, and the agreement to use the data for primary and secondary data analyses. It will be clearly stated that the participant is free to withdraw from the study at any time for any reason without prejudice to future care, without affecting their legal rights, and with no obligation to provide a reason for withdrawal.

All participants will be allowed as much time as needed to consider the information and an opportunity to question the investigator or other independent parties to decide whether they will participate in the study. Written informed consent will then be obtained by means of a participant-dated signature and a dated signature of the person who presented and obtained the informed consent. A copy of the signed informed consent will be provided to each participant. The original signed form will be retained at the study site.

There will be no participation fee nor any payment to any participant in the study.

Each participant will receive a unique number that will represent their unique ID in the study. Their blood sample will carry the same ID. The database will not include the name of the participants, as they will be deidentified, but their initials and national unique ID will be used to form a code (eg, John Doe with the unique ID 43 will have the code JD_43).

## Results

The collection of study data started in December 2021, and it is currently ongoing. Since the start of the study, 53 patients with IBD have been recruited and 50 HC were matched until the moment of this paper being published. The blood samples collected were stored according to the laboratory procedures. Data collection should end in January 2030. Intermediary analysis will be performed in April 2024 after the first 50 patients recruited go through the 12-month follow-up visit. Recruitment will continue until the number of participants in the IBD and HC groups is met (n=100 for each group).

## Discussion

### Summary

The aim of the study is to evaluate whether there is a correlation between the level of the blood biomarkers studied (SAA, Hcy, S100B, and BDNF) and cognitive scores (MOCA, MIS, FDST, BDST, TMT-A, TMT-B, DSST) in a period of 12-48 months in patients with IBD compared to HC. Previous studies have shown that patients with IBD have lower cognitive scores compared to HC [3,5,8,9,11,12,35]. We expect similar results. Multiple biomarkers have been found modified in patients with MCI but also in patients with IBD; therefore, some reliable biomarkers have been selected for analysis [19-34]. A positive correlation between worse cognitive scores and elevated serum levels is expected to be found.

### Perspectives and Clinical Implications

This study might serve as another “brick in the wall” for the question of whether the GBA is involved in the development of MCI. If the results show a correlation between the clinical onset and length of IBD and the risk of MCI development, we could rethink treatment strategies. Serum biomarkers could prove a useful tool for stratifying the risk of MCI development, as their levels can be determined for all patients and the costs are not high.

A possible link between IBD and MCI through the GBA should be further investigated at the molecular level in order to find the underlying pathogenic mechanism. According to the diagnosis criteria of AD, if we find a progression from MCI to AD in patients with IBD, perhaps a clinical study involving the detection of abnormal proteins in the cephalorahidian fluid obtained from a lumbar puncture could be performed in the future. In addition, we could expect some clinical trials involving prevention of the progression from MCI to AD if some more underlying pathogenic mechanisms are found. Furthermore, clinical trials studying the possible prophylactic treatments for the development of MCI must be performed in order to stop this invalidating disease from affecting the population.

### Strengths and Limitations

One of the biggest limitations nowadays is the fear of the hospital, a so-called “syndrome” caused by the COVID-19 pandemic. However, this is overcome by the fact that many patients require medical prescriptions monthly or at 2-3-month intervals, so they have to visit the hospital. Language will be a barrier in this study because there are many cognitive tests and questionnaires that have never been validated clinically in Romania, so we have a narrow range to pick from. Neither telephonic nor video testing is available due to language issues. Finance is also a step-back in the development of this study, but it will not limit the results, because it is a prospective and screening study.

### Risk of Bias

The risks of bias are negligible because the tests used have clear instructions and interpretations. However, there is a risk that the participants might grow impatient and not correctly complete the questionnaires or might rush them to finish them faster. To reduce the risk of bias, the participants will be taken to a silent room and enough time will be provided for them to complete the questionnaires and tests in the best-possible correctly. In addition, clinical data will be obtained before laboratory biomarkers are dosed, so we will not know the results—whether they predict the development of MCI.

### Risks and Side Effects

The risks associated with this study are the risks of normal blood collection, which is conducted anyway from time to time when patients visit the hospital for their regular blood analysis. In addition, almost all presentations at the hospital require blood sample collection; therefore, the study should not carry an additional risk. Other minor risks could be found in participants with stress or anxiety, and the fact that they will be interviewed for depression, anxiety, stress, and cognitive function might serve as an aggravating factor. Some patients might not take the tests seriously and might score worse on cognitive testing.

### Unexpected Findings

The results may be contrary to the aim of the study. To the best of our knowledge, patients with IBD have a good level of education and this might lead to better cognitive functioning. In addition, patients with IBD are younger in age, and because education is better nowadays, cognitive tests could show better cognitive functioning among well-educated participants. Furthermore, maybe some treatments that are used to control IBD serve as protective factors against MCI, or maybe other medications might be a risk factor for the development of MCI.

### Plan for Publication and Future Data Collection

The first phase of the protocol will include 50 patients with IBD and 50 HC, who will be evaluated for 12 months, and the results will be published. Furthermore, we will start applying for others grants in order to continue the clinical study. Details of the enrolled participants will be recorded in a database, and further studies will start based on the results of the first phase. Another study based on the COVID-19 questionnaire will be conducted with participants not included in this protocol (this study includes just partial results). Next, a third study on the quality of life and depression, anxiety, and stress screening will also be conducted with participants not included in this protocol. Finally, a fourth study including results from the first phase with participants not included in this protocol will be conducted.

### Conclusion

The GBA might be the link between IBD and the development of MCI associated with systemic inflammation. Mood disorders can be a prodrome for the development of cognitive dysfunction. Serum biomarkers might serve as cheap predictive factors for the development of MCI. Therapies might require adjustments in order to prevent neurological complications of IBD.

## Abbreviations

AD: Alzheimer’s disease
ADL: activities of daily living
BDNF: brain-derived neurotrophic factor
BDST: backward digit span testing
CD: Crohn’s disease
DASS: Depression Anxiety Stress Scale
DSST: Digit Symbol Substitution Test
ELISA: enzyme-linked immunosorbent assay
FDST: forward digit span testing
GBA: gut–brain axis
HBI: Harvey-Bradshaw Index
HC: healthy controls
Hcy: homocysteine
IADL: Instrumental Activities of Daily Living
IBD: inflammatory bowel disease
MCI: mild cognitive impairment
MIS: memory impairment score
MOCA: Montreal Cognitive Assessment
S100B: S100 calcium-binding protein B
SAA: serum amyloid A
SCCAI: Simple Clinical Colitis Activity Index
TMT: Trail Making Test
UC: ulcerative colitis
WHOQOL-BREF: World Health Organization Quality of Life Brief Version

## Data Availability

Study data will be available upon reasonable request from the corresponding author. The data will not be published on a public platform for ethical reasons. The pseudonymization of the participants will be removed from the database if a request for access is received.
